# Novel *BRAF* Alteration in a Sporadic Pilocytic Astrocytoma

**DOI:** 10.1155/2012/418672

**Published:** 2012-04-03

**Authors:** Sonika Dahiya, Jinsheng Yu, Aparna Kaul, Jeffrey R. Leonard, David H. Gutmann

**Affiliations:** ^1^Department of Pathology & Immunology, Washington University School of Medicine, St. Louis, MO 63110, USA; ^2^Department of Neurology, Washington University School of Medicine, P.O. Box 8111, 660 South Euclid Avenue, St. Louis, MO 63110, USA; ^3^Department of Neurosurgery, Washington University School of Medicine, St. Louis, MO 63110, USA

## Abstract

Pilocytic astrocytoma (PA) is the most frequently encountered glial tumor (glioma or astrocytoma) in children. Recent studies have identified alterations in the *BRAF* serine/threonine kinase gene as the likely causative mutation in these childhood brain tumors. The majority of these genetic changes involve chromosome 7q34 tandem duplication, resulting in aberrant *BRAF* fusion transcripts. In this paper, we describe a novel *KIAA1549:BRAF* fusion transcript in a sporadic PA tumor associated with increased ERK activation and review the spectrum of *BRAF* genetic alterations in this common pediatric low-grade central nervous system neoplasm.

## 1. Introduction

Pilocytic astrocytomas are the most common nonmalignant brain tumor in the pediatric population. Children with the Neurofibromatosis type 1 (*NF1*) inherited cancer predisposition syndrome are prone to the development of these glial cell neoplasms, such that 15–20% of affected individuals will develop gliomas involving the optic pathway, hypothalamus, and brainstem [1]. Molecular analysis of these tumors has revealed biallelic inactivation of the *NF1* tumor suppressor gene, resulting in loss of *NF1* protein (neurofibromin) expression. However, sporadic PA tumors do not exhibit mutational inactivation of the *NF1* gene, suggesting that other genetic mutations are responsible for the genesis of these histologically-identical low-grade brain tumors in the general population [2].

Over the past several years, the molecular basis for these nonsyndromic pediatric brain cancers has been elucidated with the identification of signature molecular changes involving the *BRAF* serine/threonine kinase gene. The most frequently encountered genetic alteration is a tandem duplication of the *BRAF* gene on chromosome 7q34, leading to fusion of the *KIAA1549* gene to the carboxyl terminal region of the *BRAF* gene containing the kinase domain. This molecular change has been reported in 50–65% of sporadic pilocytic astrocytoma and is more frequent in cerebellar (~80%) tumors. The majority of these alterations involve fusions between *KIAA1549* exon 16 and *BRAF* exon 9, *KIAA1549* exon 15 and *BRAF* exon 9, and *KIAA1549* exon 16 and *BRAF* exon 11 [3–9], while less common alterations include tandem duplications involving *SRGAP3* and *RAF1* or *FAM131B* and *BRAF* [10, 11]. In this paper, we describe a novel *KIAA1549-BRAF* fusion event in a sporadic pediatric pilocytic astrocytoma.

## 2. Case Presentation

**Figure 1:**
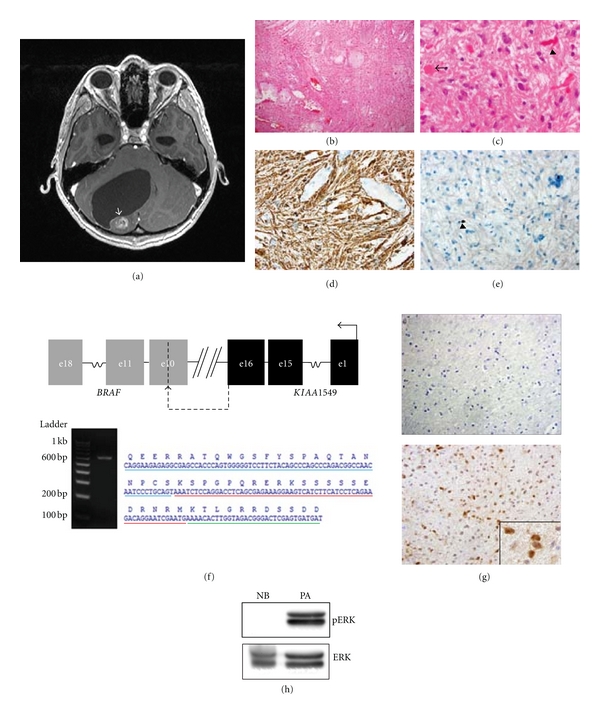
Molecular characterization of a novel *KIAA1549:BRAF* fusion alteration in a sporadic pediatric pilocytic astrocytoma. (a) Axial T1-weighted 1.5-Tesla gadolinium-enhanced MRI scan reveals a cystic lesion in the cerebellum with a peripheral enhancing nodule (arrow). Hematoxylin and eosin staining demonstrates a classic pilocytic astrocytoma with compact and loose areas (b), including Rosenthal fibers (arrow) and eosinophilic granular bodies (arrowhead) (c). The tumor is composed of cells with strong GFAP expression (d) and rare Ki-67 immunoreactivity (arrowhead; (e)). Direct amplification of RNA from this tumor demonstrates a 599 bp fragment, which creates a novel fusion *KIAA1549*:*BRAF* transcript in which exon 16 of the *KIAA1549* gene is joined to *BRAF* sequences in the middle of exon 10. The bars below the predicted amino acid sequence correspond to *BRAF* exon 10 (red), *BRAF* exon 11 (green), and *KIAA1549* exon 16 (blue) (f). Immunostaining with phospho-ERK-Thr^202^/Tyr^204^ antibodies demonstrates increased ERK activation in the PA tumor (bottom panel). Normal adult human frontal lobe (NB) from an autopsy specimen was included as reference tissue in the top panel (g). Western blot demonstrates 282-fold increase in ERK activation (phospho-ERK-Thr^202^/Tyr^204^; p-ERK; Cell Signaling Technologies, catalog no. 4370S) in the tumor (PA) relative to normal human brain (NB). Total ERK is included as internal control for protein loading (h).

The patient was a 14-year-old boy who presented with a 6-month history of headache that progressed to a two-day period of nausea, vomiting, and ataxia. Magnetic resonance imaging (MRI) at that time showed a cystic mass in the cerebellum compressing the fourth ventricle (Figure 1(a)). He was taken to the operating room where a gross total resection was performed. Neuropathological review revealed a classic pilocytic astrocytoma with alternating areas of compact and loose tissue architecture (Figure 1(b)). The compact areas were composed of piloid neoplastic cells containing numerous Rosenthal fibers and few eosinophilic granular bodies (Figure 1(c)), while the paucicellular areas were largely myxoid with scattered pleomorphic tumor cells, often containing multiple nuclei. Consistent with the glial nature of this tumor, there was diffuse and strong glial fibrillary acidic protein (GFAP) expression in the neoplastic cells (Figure 1(d)). The Ki67 labeling (proliferative) index was <1% (Figure 1(e)), and mitotic figures were not identified. Upon two-year followup, there was no evidence of recurrent tumor on MRI. To identify the molecular alteration in this pilocytic astrocytoma, RNA was extracted from a snap-frozen tumor specimen using the RNeasy mini-kit (QIAGEN), reverse transcribed, and amplified by PCR using *BRAF* and *KIAA1549* primers as previously reported [8]. Both strands of the resulting novel 599 base pair (bp) product were directly sequenced on an ABI 3730xl DNA Analyzer. In contrast to previously reported *KIAA1549:BRAF* alterations, this tumor harbored a novel fusion transcript in which exon 16 of the *KIAA1549* gene was fused to sequences within exon 10 of the *BRAF* gene (Figure 1(f)), generating a protein product in which the *BRAF* kinase domain is intact. This would result in a molecule in which the carboxyl terminal kinase domain is not bound by the amino terminal *BRAF* regulatory domain and is thus “constitutively” active, leading to downstream MEK and ERK activation. Consistent with this prediction, we found increased ERK activation using activation-specific (phospho-Thr^202^/Tyr^204^) antibodies in the tumor by both immunohistochemistry (Figure 1(g)) and Western immunoblotting (Figure 1(h)).

## 3. Discussion

The vast majority of previously reported molecular alterations in sporadic involve *BRAF* exons 9 (85% of reported *KIAA1549:BRAF* fusion transcripts) and 11 (12% of reported *KIAA1549:BRAF* transcripts) (Table 1). Similarly, all of the *FAM131B:BRAF* fusion products also included *BRAF* exon 9 sequences [11]. The current paper describes only the second *KIAA1549:BRAF* fusion event involving exon 10 [7] and is the first in which the alteration eliminates nearly half of the exon 10-encoded *BRAF* protein sequence. The inclusion of this specific genetic alteration to the growing list of *BRAF* molecular changes supports a model in which fusion events that maintain the *BRAF* open reading frame and include the *BRAF* protein sequences encoded by exons 11–18 (*BRAF* kinase domain) are potentially tumorigenic.

This proposed tumorigenicity is attributed to constitutive activation of the *BRAF* kinase domain as a result of the removal of the amino terminal inhibitory domain, leading to increased signaling to its immediate downstream effectors, MEK and ERK. Similar to other *BRAF* mutations, this novel *KIAA1549:BRAF* molecular alteration is also associated with increased ERK activity. However, the exact mechanism by which deregulated MEK/ERK activation resulting from *KIAA1549:BRAF* leads to pilocytic astrocytoma development is unclear. In this regard, several groups have shown that the expression of constitutively active (oncogenic) *BRAF* (BRAF^V600^; V600E mutation within the *BRAF* activation domain) in human astrocytes and glial progenitor cells leads to cellular senescence *in vitro *[12], and neither oncogenic BRAF^V600^ nor *RAF1* expression in mice results in glioma formation *in vivo* [13, 14]. However, forced expression of the kinase domain of BRAF^V600^, but not of wild-type *BRAF* (as exists in *KIAA1549:BRAF* fusion protein products), is transforming in primary human astrocytes *in vitro* and can induce tumors in mice *in vivo *[13]. 

NF1-associated pilocytic astrocytomas also exhibit increased ERK activation as a result of mutation loss of the NF1 tumor suppressor protein, neurofibromin. In primary mouse astrocytes, loss of neurofibromin Ras GTPase activating protein (GAP) activity leads to high levels of Ras effector (ERK, AKT) activation. However, *Nf1* genetically engineered mouse optic glioma growth is attenuated by inhibiting AKT pathway signaling [15]. In these studies, inhibition of AKT-mediated mammalian target of rapamycin (mTOR) activity using the macrolide rapamycin resulted in reduced optic glioma volume and proliferation. In light of these observations, the molecular mechanism shared by *BRAF* activation and neurofibromin loss will require further experimental investigation.

In this regard, future studies will likewise be required to determine precisely how *BRAF* activation leads to glioma formation either alone or in concert with other genetic or stromal (microenvironment) changes. Despite these seemingly contradictory experimental observations, the identification of *BRAF* as a seminal genetic alteration in pilocytic astrocytoma sets the stage for therapeutic trials aimed at restoring deregulated *BRAF*/*RAF* signaling in this common pediatric brain tumor.

## Figures and Tables

**Table 1:** Summary of reported *BRAF* fusion transcripts.

Fusion partner	*BRAF*	Number of cases	% cases
*KIAA1549* exon 16	exon 9	136	62.4
*KIAA1549* exon 15	exon 9	47	22.6
*KIAA1549* exon 11	exon 11	29	12.3
*KIAA1549* exon 18	exon 10	1	<1
*KIAA1549* exon 19	exon 9	1	<1
*KIAA1549* exon 16	exon 10*	1	<1
*FAM131B*	exon 9	3	1.4

*Current paper.
